# Sampling and Homogenization Strategies Significantly Influence the Detection of Foodborne Pathogens in Meat

**DOI:** 10.1155/2015/145437

**Published:** 2015-10-11

**Authors:** Alexander Rohde, Jens Andre Hammerl, Bernd Appel, Ralf Dieckmann, Sascha Al Dahouk

**Affiliations:** ^1^Federal Institute for Risk Assessment, Diedersdorfer Weg 1, 12277 Berlin, Germany; ^2^Department of Biology, Chemistry and Pharmacy, Free University Berlin, Takustraße 3, 14195 Berlin, Germany

## Abstract

Efficient preparation of food samples, comprising sampling and homogenization, for microbiological testing is an essential, yet largely neglected, component of foodstuff control. *Salmonella enterica* spiked chicken breasts were used as a surface contamination model whereas salami and meat paste acted as models of inner-matrix contamination. A systematic comparison of different homogenization approaches, namely, stomaching, sonication, and milling by FastPrep-24 or SpeedMill, revealed that for surface contamination a broad range of sample pretreatment steps is applicable and loss of culturability due to the homogenization procedure is marginal. In contrast, for inner-matrix contamination long treatments up to 8 min are required and only FastPrep-24 as a large-volume milling device produced consistently good recovery rates. In addition, sampling of different regions of the spiked sausages showed that pathogens are not necessarily homogenously distributed throughout the entire matrix. Instead, in meat paste the core region contained considerably more pathogens compared to the rim, whereas in the salamis the distribution was more even with an increased concentration within the intermediate region of the sausages. Our results indicate that sampling and homogenization as integral parts of food microbiology and monitoring deserve more attention to further improve food safety.

## 1. Introduction

Despite the rise of novel molecular and high-throughput detection methods, the recovery, isolation, and enumeration of bacterial pathogens in food are still primarily based on culture techniques, the current gold standard in food microbiology [1, 2]. The continuing dominance of traditional microbiological detection methods in foodstuff control is attributed to the goal to prove the absence or presence of living pathogenic bacteria, which is indispensable to assess the actual health hazard for consumers. The mere presence of bacterial DNA, which represents the target for many rapid techniques like PCR, cannot predict the risk of infection. However, the formation of visible colonies requires the successful recovery of the target bacteria out of a food matrix in a viable and replication-competent state. Thus, sample preparation is critical for the successful subsequent microbiological detection and has to be adapted to the respective food matrix [3, 4].

The initial extraction of the pathogen is usually performed by applying mechanical forces of varying magnitude to homogenize the food matrix [5, 6]. In addition to simple procedures such as vortexing or manual release, various technical solutions are commercially available. Peristaltic blenders like the Stomacher or, alternatively, the Pulsifier are probably the most prominent ones for microbiological detection [7–9]. The widely used Stomacher consists of two quickly movable paddles, which disperse the input food sample/buffer mix for enrichment via cultivation. Other homogenization methods, using beads to mill food or the application of ultrasound, might be also employed for this purpose. Such applications may be also used for the disruption of cells to release proteins or DNA for molecular detection techniques or further downstream purification processes [10, 11]. Many novel systems (e.g., FastPrep-24) also offer the possibility of adapting the homogenization device to the food matrix by adding further components like quartz sand or beads of variable sizes. The effects of these diverging approaches of sample pretreatment on cell viability and test sensitivity have been insufficiently investigated so far. Furthermore, the instructions in the highly standardized and widely accepted ISO standards for the identification of microbes in food are usually rather vague and unspecific with respect to the sampling process as well as the sample pretreatment and homogenization (in contrast to the downstream detection procedures). Thus the current use mainly depends on the availability of the devices mentioned above as well as on personal preferences and rarely considers the physical properties of the food matrix. Due to limited resources, some laboratories may entirely rely on manual homogenization or simple vortexing.

Bacterial pathogens, such as* Salmonella enterica*, might be located on the surface of a food product due to cross contamination during slaughtering in case of meat or during harvest and subsequent transport. In contrast, processed products like sausages or cheese can get contaminated inside the food product during the production process [12–14]. Little is known about the nature of the microbial burden, whether it is evenly distributed throughout the entire product or whether a microbial gradient towards the surface is present. In the latter case, an arbitrarily taken sample might cause false-negative results. Likewise, the use of different homogenization approaches for the extraction of inner microbial contamination and the microbial survival rate after the imposed shear forces have not been systematically compared and neither has the physical detachment of bacteria from the surface been evaluated [15, 16]. In case of* Salmonella* contaminated fresh produce as well as pathogenic bacteria on fish, studies indicate the significance of sample preparation [17–19].

In this study, the applicability of four different mechanical homogenizing devices (stomaching by Bagmixer 400, milling by FastPrep-24 or SpeedMill, and sonication by the Branson Sonifier, Table 1) for pathogen isolation and conventional detection by cultivation for processed and unprocessed meat products was evaluated. As a proof of principle, using* Salmonella enterica*, surface contamination was established on chicken breasts and inner-matrix contamination was established in pork sausages of soft and hard consistence. The microbial survival rate of this Gram-negative pathogen and the recovery success of each method were assessed. Additionally, the influence of sample taking on the test outcome was investigated.

## 2. Materials and Methods

### 2.1. Bacterial Strains and Growth Conditions

Exemplarily for Gram-negative Enterobacteriaceae, the* Salmonella enterica* serovar Typhimurium (*S. enterica*) reference strain DSM 11320 (DSMZ, Braunschweig, Germany) was used for all experiments.* S. enterica* was cultivated under aerobic conditions at 37°C in lysogeny broth (LB) medium. For spiking experiments, aliquots of overnight cultures were transferred into fresh LB and cultivated under rotational shaking (GFL shaking incubator 3033, 180–200 rpm) until an optical density (OD_588_) of 0.5 was reached. These cultures were serially diluted using 1% buffered peptone water (w/vol). Bacterial titers were enumerated after plating 100 *μ*L of the dilution steps on selective media (Xylose Lysine Deoxycholate (XLD) agar, Oxoid, Wesel, Germany) and incubation for 22–26 h at 37°C.

### 2.2. Spiking of Food Products

Two types of meat contamination were simulated: surface contamination and inner-matrix contamination in which the pathogen can be distributed throughout the entire food matrix. In all food samples the absence of* Salmonella* prior to spiking was confirmed according to DIN EN ISO 6579:2002 (microbiology of food and animal feeding stuffs-horizontal method for the detection of* Salmonella* spp.). Only sporadic occurrence of other bacteria such as* Serratia*,* Hafnia*, or* Citrobacter* was found.

For artificial surface contamination, chicken breasts purchased at local supermarkets (Berlin, Germany) in January 2014 were used. One or two cubical-shaped pieces of chicken breast were cut using sterile equipment and razors to obtain meat samples with a weight of 4 g (BagMixer 400, Interscience, Germany), 3 g (FastPrep-24 (MP Biomedicals, France) and sonication), and 0.15 g (SpeedMill, Analytik Jena AG, Germany). On the surface of the samples, peptone water containing* Salmonella* (volume addition of 360 *μ*L for stomaching, 270 *μ*L for FastPrep-24 and sonication, and 135 *μ*L for SpeedMill) was evenly applied to obtain spiked samples with a final bacterial load of approximately 3 × 10^5^ CFU/g, corresponding to 3 × 10^4^ CFU/mL in the homogenate. These samples were then incubated for 1 h at 4°C. Meat samples without artificial contamination were used as negative controls.

For the simulation of inner-matrix contamination, coarsely ground, smoked, or air-dried salami with a high degree of hardness and German Mettwurst (meat paste), a finely ground spreadable sausage of soft consistency, was produced in the technology facilities of the Federal Institute for Risk Assessment. These sausages, made of lean pork and bacon, were spiked under BSL-2 conditions within the production process with* Salmonella* in six different concentrations ranging from 1 CFU/g to 10^8^ CFU/g. One preparation without* Salmonella* was used as negative control. Briefly, 3.7 kg of meat (2.4 kg lean pork, 1.3 kg bacon) was mixed with 50 mL of* Salmonella* solution containing the respective pathogen concentration while being minced and flavoured (90 g nitrite salting mix, 12 g paprika, and 8 g black pepper) in an automated meat cutter (HFM Fleischereimaschinen, Germany). Smoked salamis were cured for five days at 18°C in the Bastra MC 500; German Mettwurst was cured for 2 h and afterwards allowed to ripen for six days at 18°C, before being stored at −20°C until use.

### 2.3. Homogenization Procedure and Sausage Sampling

To evaluate the efficacy of various methods to homogenize meat and meat products, four devices with diverging technical approaches were systematically compared (Table 1). The SpeedMill is a small portable milling apparatus, suitable for on-site sample processing and easily adaptable to different sample types. Likewise, FastPrep-24 is a large-volume milling tool (adaptable also to small-volume samples), which also offers a broad range of modular adaptations by adding quartz sand or beads. Stomaching was chosen because it is one of the most frequently used ways to prepare food samples. Stomaching is considered as a gentle homogenization method since heat development is marginal and the peristaltic movements of the paddles distribute the input energy on a large area, reducing potential peaks in shear forces. Finally, sonication is a rather old, but simple, method, which exerts a distinct kind of mechanical force compared to stomaching or milling.

All food samples were diluted 1 : 10 in buffered peptone water (thus 36 mL for stomaching; 27 mL for sonication and FastPrep-24; 1.35 mL for homogenization in the SpeedMill). For stomaching, samples were placed in sterile stomacher bags (BagPage 400 mL, Interscience). Homogenization was performed at the highest intensity (paddle distance 7 mm) at indicated time intervals. Milling was performed with the FastPrep-24 system at a velocity of 5 m/s (500 Watts) and with the SpeedMill (150 Watts). In case of the FastPrep-24 device, BD Falcon 50 mL tubes (BD Biosciences, Germany) were filled with three ceramic beads with a size of 6.35 mm (1/4′′ Ceramic Sphere MP). For the SpeedMill, 2 mL innuSpeed Lysis tubes E with 2.4–2.8 mm ceramic spheres (Analytik Jena) were employed. Sonication was performed under continuous cooling with circulating chilled water at the highest intensity (output power 400 Watts) in BD Falcon 50 mL tubes placed in the water-filled cup horn of a Branson Sonifier 450 (Branson Ultrasonics). An initial aliquot was taken before homogenization after vortexing for 20 s. Further aliquots were taken after 30 s and 1, 2, 4, and 8 min of homogenization. As the FastPrep-24 system requires a cooldown period of 5 min after 1 min of homogenization, food samples were cooled on ice for this time period after each minute of homogenization. All aliquots were diluted appropriately in buffered peptone water and plated twice in suitable dilution steps on selective medium. After an incubation period of 24 h at 37°C, colonies on XLD were enumerated. To monitor the accompanying flora of the food products, 100 *μ*L of samples was also plated on LB agar and cultivated under aerobic and microaerobic conditions. Ambiguous colony morphologies and sporadically observed putatively accompanying flora on XLD agar plates were analyzed by the MALDI Biotyper (Bruker, Germany), following the instructions of the manufacturer. All homogenization experiments were performed in three independent tests.

To compare the different homogenization devices for the inner-matrix contamination, whole cross sections of the sausages were used, covering all regions of the sausages. To determine the spatial distribution of* Salmonella* within the sausages, four different regions were investigated, the inner core (A), the outer rim (B), whole cross sections covering the entire area of the sausage (C), and the intermediate region (D) without outer rim and inner core. For the inner core, sausage slices were cut and circular center pieces with a diameter of approximately 7 mm were taken. For the rim, the outer 3 mm of the sausages was used. A total of 3 g out of each region was diluted 1 : 10 in buffered peptone water and homogenized via 8 min of FastPrep-24 treatment as described above. All distribution experiments were again performed in three independent tests.

### 2.4. Statistical Analyses

Two-tailed unpaired Student's *t*-test was used to evaluate the significance of the results, assuming unequal variance between the two compared groups; *P* values below 0.05 were considered as significant. All data are given as means with standard deviations.

## 3. Results and Discussion

### 3.1. Surface Contamination

**Figure 1:**
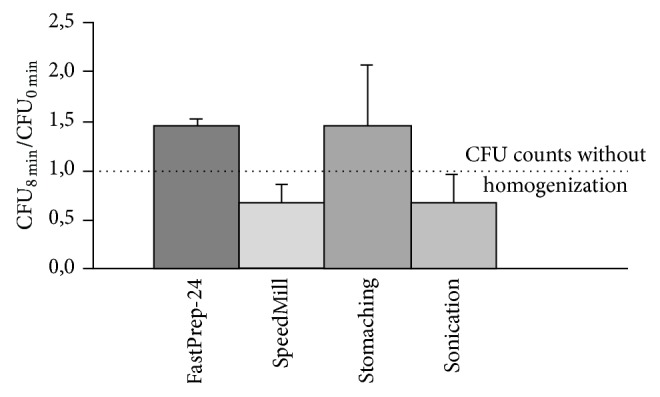
Changes in pathogen detection of chicken surface contamination after homogenization. The indicated bars express the normalized pathogen concentrations released from spiked chicken breast samples after 8 min of homogenization in relation to the CFU count after 20 s of sole vortexing.

To compare and evaluate the four different homogenization approaches (Table 1), we first determined the release of bacteria from chicken breast surfaces artificially spiked with* S. enterica*. Immediately after 20 s of vortexing, considerable numbers of* Salmonella* were already found in the medium without homogenization. However, 8 min of homogenization resulted in an increase of the number of bacteria released after stomaching and FastPrep-24 treatment, whereas sonication and SpeedMill treatment led to a decrease in pathogen recovery compared to the CFU counts obtained after an initial rinse and vortexing step (Figure 1). Independent of the applied method, there was no significant difference between the recovery rates of bacteria from homogenized and nonhomogenized samples (*P* > 0.05). However, the intermethod comparison revealed a rather good performance of both FastPrep-24 and stomaching, on the one hand, and CFU losses by sonication and SpeedMill, on the other hand. Notably, the different methods differed substantially in the degree of sample disintegration as well as in the amount of generated foam, which hampers accurate pipetting. SpeedMill, and to a lesser extent FastPrep-24, produced the greatest amounts of debris and foam, while sonication causes only a slow increase in liquid turbidity and only limited fragmentation of the food samples.

To rule out that the homogenization procedure by itself exhibits a negative effect on the viability of* S. enterica*, thereby explaining the inferior results of SpeedMill and sonication, we investigated the effects on pure* Salmonella* cultures, diluted in buffered peptone water. No CFU loss was found for SpeedMill, even after 8 min of homogenization; in contrast, sonication of pure cultures for 8 min diminished the CFU count by roughly one-fourth, in accordance with the reduction seen for the surface contamination of chicken breast. This might indicate that the inferior performance of sonication is indeed a result of slow bacterial killing while SpeedMill treatment does not affect bacterial viability in pure cultures but seems to be unable to separate bacteria efficiently from the food surface. The results obtained for* S. enterica* are probably valid for other disease-causing members of the Enterobacteriaceae like* Escherichia coli* or* Yersinia* spp. In addition, it is likely that smaller bacteria like* Campylobacter* or Gram-positive pathogens like* Listeria monocytogenes* are also not affected in their viability after extensive homogenization because mechanical shear forces in general are more harmful for larger objects than for smaller ones. However, smaller beads and sphere materials other than ceramic might exhibit much more unfavorable shear forces. In accordance with this assumption, the manufacturers of FastPrep-24 and SpeedMill, MP Biomedicals and Analytik Jena, recommend the use of considerably smaller beads to lyse bacteria for subsequent molecular detection methods.

In contrast to inner-matrix contamination, for which only scarce information is available so far, it has been reported that, for surface contamination, extended periods of stomaching and other homogenization methods did not significantly enhance the detection of the pathogen compared to simple rinsing procedures [11, 15, 20]. It was suggested by Sharpe and others that this might be the result of a “mass action” effect which prevents a higher pathogen release after equilibrium between the liquid phase and the food surface has been reached. Although this hypothesis has not yet been proven, it illustrates that longer homogenization periods and harsh homogenization methods are not necessary for surface contamination; instead soaking, hand-massaging, or the use of swabs for those food products might be equally applicable or even better procedures [6, 18, 21, 22].

### 3.2. Inner-Matrix Contamination

**Figure 2:**
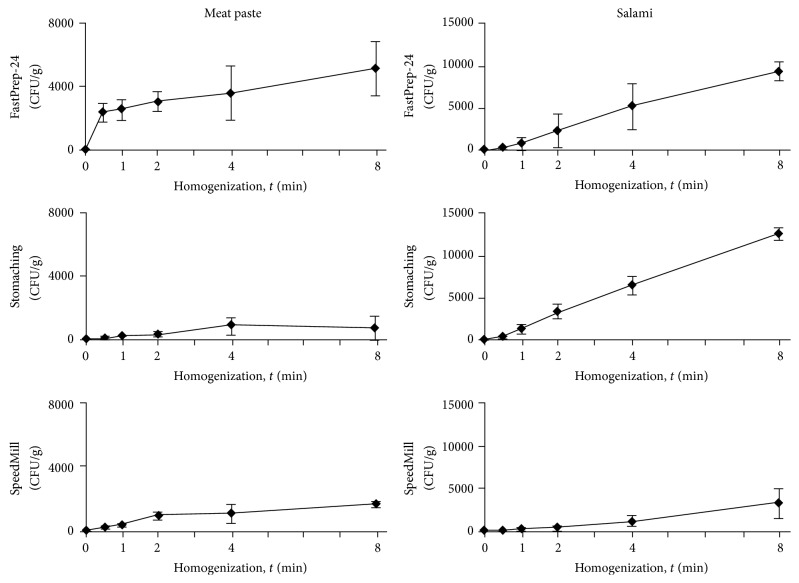
Homogenization of inner-matrix contamination. Release of* Salmonella* from whole cross sections of internally contaminated sausages after pretreatment by FastPrep-24, stomaching, and SpeedMill for 0, 30 s and 1, 2, 4, and 8 min was monitored.

To elucidate the efficiency of mechanical disruption for the detection of inner-matrix contamination, two sausages with an artificial* Salmonella* contamination were produced. During the production, ripening and storage of the spiked sausages, the number of pathogens in the sample dropped by 3 to 4 log units. In the salamis, slightly higher concentrations of* S. enterica* compared to the meat paste were found despite being spiked in equal amounts, which might be a result of the shorter curing and ripening period (in total, five days for the salamis and six days for the meat paste). For the homogenization experiments, sausages primarily spiked with 10^8^ CFU/g were chosen to yield sufficient CFU counts for an adequate interpretation. In contrast to surface contamination, the two sausage types showed substantial differences in terms of pathogen release (Figure 2). For the soft, finely minced meat paste, FastPrep-24, the large-volume milling device, showed a superior performance, extracting seven times more pathogens after 8 min than stomaching (Figure 2; left column). The low-volume SpeedMill enabled intermediate extraction success, whereas sonication, which was unable to substantially break up the sausage matrix, yielded no CFUs at all (data not shown). Interestingly, independent of the system, the longer the homogenization period was chosen, the higher the number of detectable CFUs was, although a burst of pathogen release was already found after 30 s of homogenization. Simple vortexing and rinsing procedures (before homogenization; *t* = 0) yielded no CFUs, demonstrating that this procedure is only suitable for surface contamination. For the hard, coarsely ground salami, FastPrep-24 treatment and stomaching showed a comparable release of* S. enterica* (Figure 2; right column). In contrast to the meat paste, no initial burst of pathogen release was detected. Instead, after a short lag phase a continuous, nearly linear release rate of bacteria was measured. After shorter homogenization periods, remarkably less bacteria were extracted than after longer treatment; for example, the number of extracted bacteria was 10-fold less after 1 min compared to 8 min. Similar to the meat paste, SpeedMill treatment of the salamis was rather ineffective and sonication was not able to homogenize the matrix effectively. In both sausages the amount of accompanying flora was marginal, indicating that the influence of other bacteria did not play a major role. A third sausage type, smoked salami, showed results similar to the air-dried salami. However, due to the very low* Salmonella* concentration after the smoking process (data not shown), a statistically reliable comparison was not possible.

For routine examinations of food products, homogenization is usually performed for a rather short duration (e.g., 1-2 min) to save time and by using soft mechanical treatment (stomaching or blending at low intensities) to avoid loss of bacterial viability. However, the conventional mild sample preparation is not necessarily preferable because the results in Figure 2 show that longer treatments and harsher conditions are beneficial to determine inner-matrix contamination and do not affect the bacteria. Interestingly, stomaching yielded significantly different results for the salami and the meat paste. The inferior performance of stomaching for* Salmonella* extraction out of the meat paste may result from the very high reduction ratio and, thus, a very small meat particle size of the meat paste. The meat was spiked during the mincing process and* Salmonella*, therefore, sticks to the surface of these particles. In the coarsely ground salami, the meat particle size during the spiking procedure was much bigger and as a consequence, in comparison to the meat paste, the accessible surface area was much smaller. To transfer the pathogen to the dilution medium, it is necessary to bring the pathogen in contact with the liquid phase by homogenizing the solid matrix. Consequently, harsh mechanical treatments like the FastPrep-24 system, which enable the rapid exchange between the particulate solid phase and the liquid phase, might be much more effective for finely minced sausages (e.g., meat paste) with a high particulate surface area, whereas, in case of more granular matrices with small overall surface areas, FastPrep-24 and stomaching perform similarly. In line with these considerations, a recent study with tomatoes, internally contaminated with* S. enterica*, showed that a short, but harsh, blending step was more effective than a longer stomaching treatment [19]. It would be interesting to evaluate whether suggested alternatives to stomaching like the Pulsifier, which was reported to perform equally for surface contamination while generating less debris [8, 9, 23], provide different results.

### 3.3. Analysis of Inner-Matrix Pathogen Distribution

**Figure 3:**
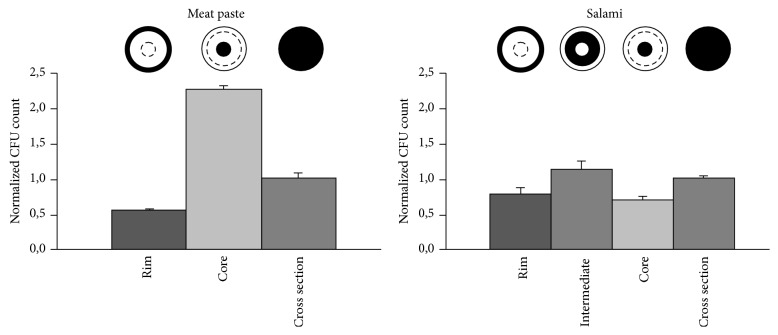
Pathogen distribution within meat paste and salami. The schematic drawings on top of the bars indicate the examined region of the sausages (black). Pathogen concentrations were determined after 8 min of FastPrep-24 treatment and are given in relation to the total concentration in whole cross sections.

Not only homogenization but also the region of sampling might influence the detection of a pathogen. Although the pathogen has been evenly distributed throughout the entire meat mass during the production process, it is possible that this even dispersal might change during the sausage ripening. Therefore, we divided the sausages into different regions, covering the outer rim, the core, and whole cross sections of the food products, and determined the concentration of* S. enterica* in each region (Figure 3). For homogenization, the FastPrep-24 treatment was chosen because this system had shown a good performance in both sausage types. The core of the meat paste contained up to fourfold higher concentrations of* Salmonella* than the outer rim, demonstrating a sharp gradient towards the center of the sausage matrix. In contrast, the distribution of* S. enterica* in the salami was relatively homogenous and no bacterial enrichment in the core was identified. However, the concentration of* Salmonella* was slightly increased within the intermediate region of this sausage type compared to rim and core. Further investigations are necessary to determine whether the pathogen distribution within the meat paste is based on the production process or on specific parameters like rigidity, water content, ingredients, or grain size of the meat.

Standard operation procedures are rather vague in terms of sample taking; in general they require “representative” samples, which can be interpreted as whole cross sections in case of sausages. However, our study shows that the assumption of a homogenous pathogen distribution within the matrix is not necessarily realistic. For the meat paste, we identified surprisingly high differences in pathogen load, presumably resulting from a prolonged survival in the sausage core, which might be a result of a different water activity or pH, both major factors for pathogen inactivation [24, 25]. Different types of sausages or in general any food product with a suspected inner-matrix contamination (thus including not only meat products but also fresh produce or dairy products) might have a characteristic distribution (and survival) pattern for a particular bacterial species, including the formation of bacterial aggregates [26–29]. Thus, it might be worthwhile to elucidate the pathogen distribution in other food products, since a more sophisticated and risk-based sampling of food regions with higher bacterial loads might enable better detection limits.

## 4. Conclusions

The results presented in this work demonstrate the pivotal role of sampling and homogenization for the reliable detection of pathogens in specific food products. It becomes evident that the general lack of precise advices regarding sample pretreatment might be responsible for considerable interlaboratory differences in pathogen detection. This not only is important for microbiological investigations, but also might be suitable as a general reference point for other whole-cell detection methods, for example, fluorescence* in situ* hybridization [30]. However, choosing the appropriate homogenization device should consider not only the efficiency of detection, but also the ease of handling, costs, and high-throughput capabilities. Summing up, to enable improved pathogen detection methods, standardized and harmonized sample preparation protocols are needed for different food matrices.

## Figures and Tables

**Table 1:** Properties of the chosen homogenization devices.

Method	Stomaching (Bagmixer 400)	FastPrep-24	SpeedMill	Branson Sonifier 450
Principle	Blending by movable paddles	Bead-mediated milling	Bead-mediated milling	Sonication

Handling	+	+	+	+/−

Portability and on-site usage	−	−	+	−

Adaptability to different matrices	+/−	++^∗^	++^∗^	+/−

Current usage for detection by cultivation	++	−	−	−

Parallel sample preparation	−	+ (2–48)^∗∗^	+ (2–20)	−

Suitability for high volumes^∗∗∗^	++ (<400 mL)	+ (<50 mL)	− (<2 mL)	+ (<50 mL)

Available volume range^∗∗∗^	+/−	+	+/−	+

Avoidance of heat generation	+	+/−	+/−	+/−

Performance in this study				
Surface contamination	+	+	+	+
Inner-matrix contamination	Variable	+	+/−	−

++: excellent, +: good, +/−: ambiguous, and −: poor.

^∗^Various matrix-specific kits and beads for sample preparation are commercially available.

^∗∗^The parallel preparation of 48 samples is only possible for volumes smaller than 2 mL. Two samples can be homogenized simultaneously for the highest volume input.

^∗∗∗^Exact volumes depend on the sizes of the used bags, BD Falcon tubes, and lysis tubes.
